# Intention to Screen for Cervical Cancer in Debre Berhan Town, Amhara Regional State, Ethiopia: Application of Theory of Planned Behavior

**DOI:** 10.1155/2020/3024578

**Published:** 2020-03-19

**Authors:** Tomas Getahun, Mirgissa Kaba, Behailu Tariku Derseh

**Affiliations:** ^1^School of Public Health, College of Health Sciences, Addis Ababa University, Addis Ababa, Ethiopia; ^2^Department of Public Health, College of Health Sciences, Debre Berhan University, Debre Berhan, Ethiopia

## Abstract

**Background:**

Cervical cancer is a major public health problem in many developing countries. Despite the value of screening to prevent morbidity and mortality from cervical cancer, little available literature shows early detection and treatment to be limited in Ethiopia. The aim of this study was to determine the magnitude of and identify factors associated with women's intention to screen for cervical cancer using the theory of planned behavior.

**Methods:**

A community-based cross-sectional study design supplemented with a qualitative approach was employed. Using multistage sampling, a total of 821 women were used in the study. An interviewer-administered survey questionnaire was used to collect quantitative data, whereas purposively selected 12 female health care providers were included in in-depth interviews. Descriptive statistics and simple and multiple binary logistic regression analysis were used to determine the magnitude of women's intention, identify associated factors, and explore barriers for intention to cervical cancer screening among Debre Berhan women, Ethiopia. The statistical association was determined at a *P* value of less than 0.05. Moreover, thematic analysis was used to search the hindrances of women's intention to screen for cervical cancer.

**Results:**

The median age of women who participated in this study was 39 years with IQR of 35 to 42 years. Three hundred sixty-one (361, 45.3%) of women had an intention to screen for cervical cancer within three months from the date of the interview. Positive attitude towards cervical cancer screening (AOR = 6.164; 95% CI: 4.048, 9.387), positive subjective norm (AOR = 2.001; 95% CI: 1.342, 2.982), and higher perceived behavioral control (AOR = 7.105; 95% CI: 4.671, 10.807) were predictors of the women's intention to screen for cervical cancer. The qualitative finding revealed that women did not like to be screened for cervical cancer because they thought that procedure pinch the cervix and it may result in perforating the uterus that would expose them for infertility. In addition, the qualitative findings supported quantitative results, where the constructs of the theory of planned behavior play an essential role in the betterment of women's intention.

**Conclusion:**

This study showed that women's intention to screen for cervical cancer was low. Positive attitudes towards cervical cancer screening (CCS), subjective norms, and perceived behavioral control were predictors of women's intention to screen for cervical cancer. Thus, efforts should be exerted to improve the attitude of women involving influential people, which could improve women's intention for cervical cancer screening. Moreover, behavioral change communication focusing on the constructs of the theory of planned behavior is crucial.

## 1. Background

Globally, cervical cancer is the second most common type of woman cancer with an estimated 530,000 new cases and 270,000 deaths every year [1]. In developing countries, it is a major public health problem where 85% of new cases and 87% of deaths take place. In these countries, it is responsible for around 12% of all cancer cases and 7.5% of all cancer-related deaths [2].

In Ethiopia, cervical cancer is the second common type of cancer among women. In 2016, there were an estimated 29.43 million women aged 15 and above who are at risk of developing cervical cancer [3]. In the country, there were an estimated 7,095 new cases of cervical cancer and 4,732 deaths occur per year [3]. Moreover, health facility-based studies showed that the annual increment of new cases was detected in the last decade [4]. Unlike other reproductive organ cancers, cervical cancer is a preventable disease. Cervical cancer screening is the most effective and efficient intervention that can prevent about 80% morbidity and mortality from the disease; despite this fact, the screening coverage is 5% in developing countries [5].

In resource-limited countries including Ethiopia, mainly because of the poor cervical cancer screening service utilization, the majority of the patients are diagnosed at an advanced stage. Treatment at the advance stage often involves multimodality including chemotherapy, radiotherapy, and surgery and has markedly reduced survival rate. Besides, patients could be at an increased risk of infertility and pregnancy outcomes might be affected in such a way that low birth weight may be encountered [6, 7]. The barriers for cervical cancer screening services underutilization include personal barriers (lack of knowledge about the importance of screening, misconceptions, and negative health beliefs) [8, 9] and community and health system structural barriers such as lack of community support [10] and inaccessibility of the screening services [11, 12]. Recently, the government of Ethiopia is increasing the availability of cervical cancer screening service; nonetheless, recent studies in the country reported utilization of screening service is very low [5, 13]. Thus, investigators expected to search for factors associated with low cervical cancer screening service utilization among age-eligible women.

**Figure 1:**
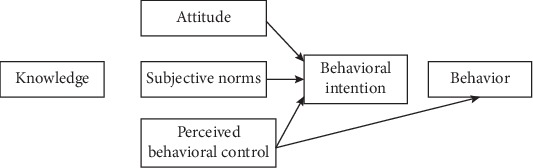
Diagram illustrating the theory of planned behavior (http://www.valuebasedmanagement.net/methods_azjen_theory_planned_behaviour.html).

Considering community health needs, concerns and their decision-making process are imperative to understand the health-seeking behavior towards any health-related conditions. Several theoretical frameworks or models have been developed that could help to explain individual or community health-seeking behavioral changes [14]. The theory of planned behavior (TPB) is one of the most commonly used frameworks to understand and predict health-related behaviors (Figure 1). The theory of planned behavior includes three main factors that can predict the intention to perform a particular behavior: attitude, subjective norm, and perceived behavioral control towards the behavior. Knowledge of these predictors is believed to be important to develop effective health promotion interventions including intention and improve the attendance rate of cervical cancer screening [15].

Despite the fact of poor utilization of cervical cancer screening, previous studies on an intention to screen for cervical cancer are limited in Ethiopia. Thus, the main purpose of this study was to assess women's intention to screen for cervical cancer and identify associated factors in Debre Berhan town, Ethiopia, based on the theoretical framework of the theory of planned behavior. Moreover, this study is aimed at exploring the barriers qualitatively influencing women's intention to screen for cervical cancer from health care providers' perspective. The study findings also may fill the gap in the existing knowledge, help policymakers when they make decisions that facilitate intention to screen for cervical cancer, serve as an important tool to develop comprehensive cervical cancer prevention programs to increase utilization of the screening services, and consequently decrease morbidity and mortality from the disease.

## 2. Methods

### 2.1. Study Design and Study Area

Community-based cross-sectional study designs using both quantitative and qualitative methods were adopted from July 1 to 30, 2017. The study was conducted in Debre Berhan town, North Shoa Zone, Amhara Regional State of Ethiopia. Debre Berhan town is located 130 km in the North-East direction from Addis Ababa, Ethiopia. Based on the projection of the 2007 census, the population of the town in 2016/17 was 103,422 (46,525 male and 56,897 female). Administratively, Debre Berhan town is subdivided into 9 urban and 5 rural kebeles (public administration units). In the town, there is one governmental referral hospital, one private general hospital, three health centers, two governmental clinics, and 12 private clinics and there are also 9 urban and 5 rural health posts. Debre Berhan health center and Debre Berhan referral hospital are currently providing cervical cancer screening services since 2016 for 30 to 49 years old women [16].

### 2.2. Sample Size Determination and Selection of Participants

For the quantitative part, the sample size for the study was determined using a single population proportion formula. Assuming the proportion of women who had the intention to screen for cervical cancer at 50% due to the absence of relevant previous study on the topic, 95% confidence level, and 5% margin of error, the sample size was determined at 384. Moreover, considering a 10% nonresponse rate and due to the multistage nature of our sampling technique, we multiplied the sample size by the design effect of 2 and hence the total sample size was 844 women. To supplement the quantitative result, we also recruited twelve female health care providers purposely and participated in in-depth interviews.

According to Ethiopian guidelines for the prevention and control of cervical cancer (9), women (aged 30–49) who are a permanent resident (had been living for at least six months) in Debre Berhan town were included in the study by using a multistage sampling technique. There are 14 kebeles (9 urban and 5 rural) in Debre Berhan town. Four urban and three rural kebeles were selected randomly by using the lottery method. The total sample size (844) was distributed to each selected kebeles based on proportion to the size of the population in the respective kebeles. Households for the study were identified following a systematic random sampling technique. If there were more than one eligible woman in the selected households, only one was chosen by the lottery method. If there were no eligible women or not available after three visits, a woman in the next nearest household was enrolled. For the qualitative part of the study, cervical cancer screening service providers from rural Kebeles were selected and enrolled purposively in consultation with the head of selected health facilities and participated in in-depth interviews.

### 2.3. Data Collection Tools and Procedures

The structured questionnaire prepared in English was adapted from a previous study [17]. The questionnaire went through translation to Amharic (local language) and back translated to English to ensure consistency. The survey held to measure women's intention to screen for cervical cancer, sociodemographic characteristics, knowledge about cervical cancer, and attitude towards cervical cancer screening, subjective norms, and perceived behavioral control on cervical cancer screening. Ten questions that were adopted from the previous study [17] helped to determine cervical cancer knowledge. The questions contain items about risk factors for getting the disease, symptoms, prevention, treatment, recommended interval, and techniques of screening.

The TPB constructs were adopted from the standard tool [18] that was developed based on the theory of planned behavior [15]. Thirteen items adapted measured the three constructs (attitude towards CCS, subjective norm about CCS, and PBC of CCS). All items' responses had unipolar scales of 1 to 5. Attitudes towards CCS were measured by four items, and the items had high internal reliability with Cronbach *α* = 0.9. The attitude was dichotomized into positive and negative; respondents who scored above the mean attitude value were considered to have a positive attitude towards CCS, and respondents who scored below the mean attitude value were considered to have a positive attitude towards CCS. Subjective norms were measured by three items, and these items had moderate internal reliability with Cronbach *α* = 0.7. Respondents who scored above the mean subjective norm value were considered to have positive subjective norms, and respondents who scored below the mean subjective norm value were considered to have positive subjective norms on CCS. PBC on CCS were measured by four items, and the items had high internal reliability with Cronbach *α* = 0.9. Respondents who scored above the mean PBC value were considered to have higher PBC on CCS, and respondents who scored below the mean PBC value were considered to have higher PBC on CCS. Intention to screen for cervical cancer was determined by two items. The overall intention score was determined by computing the mean score. The woman said to have an intention to screen if she scored above the mean intention score and no intention if she scored below the mean intention value.

The data was collected by 6 female diploma midwifery nurses who got training related to cervical cancer from another district health facility through face-to-face interviews. The principal investigator (PI) trained data collectors and supervisors for two days prior to data collection. The interviews were performed in the participants' own homes confidentially. To check the understandability of the questions, the questionnaire was pretested among 42 women in Debre Sina town, North Shoa zone, with the assumptions of similar conditions with the study area. Then, after the pretest, the possible modification was made on the tool. Trained supervisors daily supervised data collectors and checked the completeness and consistency of the responses on a daily basis. Before data analysis, the data was cleaned and cross-checked.

A semistructured checklist adapted for in-depth interviews (IDI) helped to generate evidence on women's attitudes, norms, and misconceptions towards the intention to screen for cervical cancer. The reasons to include female HCPs were women prefer to ask them about issues related to gynecological-related problems including cancer. In addition, females at different health care system levels are expected to create awareness to the community, motivate women to go through screening, and counsel and perform cervical cancer screening [5, 13]. Thus, female HCPs' views help to explain the influence of attitude, social pressure, and sense of control on women's intention to screen for cervical cancer, and finally, participants were asked about their suggestion to improve intention to screen for cervical cancer. The principal investigator conducted all the interviews in a private room, and on average, each interview took 40 minutes. Information saturation was achieved after 10 interviews, but an additional two females were interviewed to make sure the data saturation, and there was no new information obtained.

### 2.4. Data Processing and Analysis

The quantitative data were entered and cleaned using EpiData software version 3.1 and exported to SPSS version 20 for statistical analysis. To describe the data descriptive summaries, frequency tables and different graphs were used. The association of each independent variable with the outcome variable was examined by performing a simple logistic regression analysis. Collinearity diagnostics were computed, and no multicollinearity was found among constructs of the theory of planned behavior variables. Multiple logistic regression analysis was computed to control the effect of possible confounding and interaction of factors. Variables with *P* value < 0.2 during the simple logistic regression analysis were included in the final model using the enter method. For the statistical tests, *P* value ≤ 0.05 was considered as statistically significant. Adjusted odds ratio (AOR) and 95% confidence intervals (CI) were constructed to measure the strength of association between the dependent and independent variables.

The theory of planned behavior was employed to construct exploratory themes for in-depth interviews. The themes explored individual and community constructs of behavioral intention towards cervical cancer screening. These themes were 4: level of attitude, subjective norms, perceived behavior control for women's intention to screen for cervical cancer, and the possible recommendation which are used to improve intention to screen. The qualitative data was analyzed manually by the principal investigator through thematic analysis. First, the audio record was fully transcribed to a word and then translated into English. From the transcribed data, codes were created. Based on the pattern of the responses, a set of categories was identified and each category was placed in their corresponding theme. Finally, the finding was discussed by complementing with the quantitative result.

## 3. Results

Results from the quantitative and qualitative data were reviewed to pursue support, to clarify results from the quantitative research, and to extend the breadth of our understanding about women's intention to screen from cervical cancer.

### 3.1. Sociodemographic Characteristics

A total of 821 women were interviewed in the study making the response rate of 97.7%. Out of the total participants, 668 (81.4%) were urban residents. Around 311 (37.9%) of respondents were in the age group of 35-39 years; the median age was 39 years with the interquartile range of 35 to 42 years. Majority 717 (87.3%) of the respondents were Orthodox Christian, and 713 (86.8%) were Amhara in their ethnic group. About half 406 (49.5%) of them had an educational level secondary school and above, and 471 (57.4%) was the housewife. Regarding marital status, 662 (80.6%) of them were married and 662 (80.6%) of them had 1-4 children (Table 1). Twelve female health care providers were enrolled and participated in in-depth interviews.

### 3.2. Intention to Screen for Cervical Cancer

Among the total of 821 respondents, only 24 (2.9%) of them reported that they have ever been screened for cervical cancer in the past five years. The mean value of intention score was 5.3, and 361 (45.3%) of the respondents scored above the mean, then considered to have an intention to screen for cervical cancer within the next three months.

### 3.3. Factors Associated with Intention to Screen for Cervical Cancer

The result of multiple logistic regression analysis revealed that attitude towards cervical cancer screen (CCS), subjective norms, and perceived behavior control (PBC) on CCS had a significant positive association with intention to screen for cervical cancer (Table 2). The qualitative findings also supported these quantitative results. This part was thematically analyzed into four parts: women's attitude towards cervical cancer screening, subjective norms, perceived behavior control, and health care provider's recommendation to improve women's intention to screen for cervical cancer.

Respondents who had a positive attitude towards CCS were 6 times (AOR = 6.164; 95% CI: 4.405, 9.387) more likely to have the intention to screen for cervical cancer than respondents who had a negative attitude towards CCS. This result is supported by a key informant interview. One respondent exemplified in such a way that (IDI_1):

“Most women did not like to be screened for cervical cancer since they thought the screening procedure pinches the cervix and it may result in perforating the uterus that would expose them for infertility.”

Furthermore, respondents who had positive subjective norms were 2 times (AOR = 2.001; 95% CI: 1.342, 2.982) more likely to have intention compared to those with a negative subjective norm. As indicated by another female health care provider, support from the community could increase the intention of women. For instance, one participant explained that (IDI_5):

“Women would feel that they would be misunderstood by the community if they tried to seek the screening services: if someone saw them while screening for cervical cancer, they would think why this woman was screening. She was married and leave with her partner and they would think that as she has a problem.”

Likewise, respondents who had higher PBC were 7 times (AOR = 7.105; 95% CI: 4.671, 10.807) more likely to have intention than respondents with lower PBC (Table 2). This can be explained by working time as indicated by respondents (IDI_2):

“The working time for cervical screening clinics was not convenient for employed women. With their additional household responsibilities they had, their chance to go for screening was during the weekend so, unavailability of the services during this time will have an influence on the cervical screening of those women.”

### 3.4. Suggestions to Improve Women Intention to Screen for Cervical Cancer

The participants of this study recommended several ways to improve women's intention to screen for cervical cancer. They suggested creating awareness to the husband, improving the community participation, and improving the availability of the screening services. Health education should be provided for influential people regarding cervical cancer and the importance of screening.

## 4. Discussion

This study showed that the prevalence of women's intention towards cervical cancer screening was 45.3%. Positive attitudes towards cervical cancer screening, positive subjective norm, and higher perceived behavioral control were factors that affected women's intention to screen for cervical cancer. Moreover, the qualitative findings corroborate that constructs of the theory of planned behavior play a significant role in the improvement of women's intention to cervical cancer screening.

The result of this study showed that only 45.3% of respondents had the intention to screen for cervical cancer in three months from the date of data collection. This finding is consistent with a study conducted on the Ethiopian women army which showed intention for cervical cancer screening at 43.4% [19]. However, the result is considerably lower than studies conducted in Uganda [20] and Malawi [21], where 63.0% and 57.2% of the respondents intended to screen for cervical cancer, respectively. The difference might be due to variation in access to information and screening services. Most of the respondents of the former studies heard about cervical cancer and screening services introduced earlier in those countries. The qualitative findings recommended several ways to improve women's intention to screen for cervical cancer. They suggested creating awareness for women, improving community participation, and improving the availability of the screening services. They also added that health education should be provided for women regarding cervical cancer and the importance of screening. One midwife-nurse mentioned the importance of awareness creation to improve women's motivation to screen for cervical cancer. She explained (IDI_4): Health information through Mass-medias such as Televisions, and radio and printed materials about cervical cancer including its risk factors, symptoms, ways of prevention, and screening methods would change the women's myths and misconception that would improve their service utilization.

More importantly, another key informant interview participant suggested that health care provider recommendations would motivate women to attend cervical cancer screening services. Explaining (IDI_6): Health care workers' encouragement is an effective way of enhancing women's intention to undergo CCS since most of the time the women trust what health professionals said.

This study also revealed that attitude towards cervical cancer screening was significantly associated with intention to screen for cervical cancer. Respondents who had a positive attitude towards cervical cancer screening were 6 times more likely to have intention than those who had a negative attitude. A similar result was reported from a community-based cross-sectional study in China which identified attitude towards cervical cancer screening as the most significant factor that affects intention [22]. The influence of attitude on the intention to screen for cervical cancer was explained by fear of the procedure. Fear of the screening procedure could be a barrier to women's intention to screen for cervical cancer. The study participants elucidated that women's belief in screening for cervical cancer would be painful. One of the midwives interviewed explained: the use of speculum during the procedure creates fear for clients. But after the examination, most of the time, they say: I thought it would be painful, but it is not. I did not feel anything.

As reported from a study conducted in Singapore [23] and rural Tanzania [24], the present study result revealed that subjective norms had a significant association with intention to screen for cervical cancer. This result was supported by the qualitative findings of this study. The HCP interviewed indicated family members and friends play an important role in women's decision to health seeking as they are the main source of advice and information. Further, the participants indicated that support and encouragement from husbands would influence women's decision to screen for cervical cancer. Given the influence of these groups on women's health-seeking behaviors, intervention to increase intention to screen for cervical cancer should target them, especially husbands. Hence, education and awareness creation on CCS should be given for those influential people so they would be supportive of women screening. Study participants interviewed gave emphasis to the influence of husbands for women decisions to be screened for cervical cancer. One respondent reported that (IDI_10): In our community, you might not make decisions by yourself, you have to discuss with your husband before you decide to do something including screening for cervical cancer. If you did not listen, you would be in conflict with your husband and it may also result at the end of your relationships.

Thus, study participants suggested that husband involvement and awareness creation to the community would improve the support to women for screening. One participant explained the importance of educating husbands to improve women's intention in cervical cancer screening (IDI_12): If husbands know the importance of screening they would support and motivate their wife to undergone CCS. So, husband involvement during motivational talks would improve the motivation for screening services utilization.

Women's perceived behavior control has been recognized to influence significantly their intention and behavior on various health conditions [25]. In this study, PBC on CCS had a significant association with intention to screen for cervical cancer. Respondents with higher PBC were 7 times more likely to have the intention to screen for cervical cancer than the respective referent group. A similar association was reported from a study conducted among Singaporean women [26].

On top of previous studies, the qualitative finding suggested the importance of PBC on women's intention; it is worth to consider improving women's sense of control and confidence by informing as the screening services provided free of cost in public health facilities and by improving the accessibility of the screening services. Moreover, women from the rural area should be organized in different income-generating activities which support their capability for daily expenditure. One study participant explained this in such a way that (IDI_9): Rural women came from areas that could be as far as 5 kilometer from the health institutions providing the screening services. It would be difficult for those women to cover the transportation cost because those women did not have their own income.

In contrast to previous studies [27, 28], this study revealed that sociodemographic characteristics and women's intention to cervical cancer had no significant association. This insignificant association could be due to the mediating effects of TPB's primary constructs (attitude, subjective norm, and PBC). The TPB suggests, “All external factors, such as demographics, or personality characteristics of the actor, the nature of particular behavior under investigations, or situational variables can affect intentions only if they influence the attitudinal or normative components or their relative weights” [29]. On the other hand, this result was in line with previous TPB-based studies which revealed no significant effect of sociodemographic characteristics on the intention to screen for cervical cancer [30].

### 4.1. Strength and Limitation of the Study

We identified different factors associated with women's intention to screen for cervical cancer among Debre Berhan town and showed that the constructs of the theory of planned behavior have a significant effect on intention. The use of a mixed-method approach makes these findings stronger and contributes to evidence-based practice. On the other hand, social desirability bias cannot be ruled out as some respondents may give a response in a positive way. But as much as possible, enumerators explained to study participants to provide a genuine response.

## 5. Conclusions

This study underlines that women's intention to screen for cervical cancer was low. Positive attitudes towards cervical cancer screening (CCS), subjective norms, and perceived behavioral control were predictors of women's intention to screen for cervical cancer. Thus, efforts should be exerted to improve the attitude of women involving influential people who could improve women's intention for cervical cancer screening. Moreover, behavioral change communication focusing on the constructs of the theory of planned behavior is crucial.

## Figures and Tables

**Table 1:** Sociodemographic characteristics of respondents, Debre Berhan town, Ethiopia 2017 (*n* = 821).

Characteristics	Frequency (%)
*Residence*	
Urban	668 (81.4)
Rural	153 (18.6)
*Age in years*	
30 to 34	168 (20.5)
35 to 39	311 (37.9)
40 to 44	225 (27.4)
45 to 49	117 (14.3)
*Religion*	
Orthodox	717 (87.3)
Protestant	68 (8.3)
Muslim	28 (3.4)
Catholic	8 (1.0)
*Ethnic group*	
Amhara	713 (86.8)
Oromo	72 (8.8)
Tigray	27 (3.3)
Gurage	9 (1.1)
*Educational level*	
No formal education	177 (21.6)
Primary	238 (29.0)
Secondary and above	406 (49.5)
*Occupation*	
Housewife	471 (57.4)
Government employee	163 (19.9)
Private employee	76 (9.3)
Farmer	111 (13.5)
*Marital status*	
Single	67 (8.2)
Married	662 (80.6)
Divorced	53 (6.5)
Windowed	39 (4.8)
*Number of children*	
No child	111 (13.5)
1 to 4	679 (82.7)
5 and above	31 (3.8)

**Table 2:** Simple and multiple logistic regression analysis of factors associated with intention to screen for cervical cancer, Debre Berhan town, Ethiopia, 2017.

Variables	Intention		COR (95% CI)	AOR (95% CI)
	Yes, *N* (%)	No, *N* (%)		
*Age group*				
30 to 34	81 (18.6)	85 (23.5)	1	1
35 to 39	154 (35.3)	146 (40.4)	0.903 (0.618, 1.32)	1.329 (0.787, 2.244)
40 to 44	132 (30.3)	89 (24.7)	0.643 (0.428, 0.964)^∗^	0.943 (0.534, 1.664)
45 to 49	69 (15.8)	41 (11.4)	0.566 (0.346, 0.926)^∗^	0.697 (0.35, 1.387)
*Religion*				
Orthodox	371 (85.1)	326 (90.3)	1	1
Protestant	42 (9.6)	24 (6.6)	0.65 (0.385, 1.097)	0.81 (0.39, 1.684)
Others	23 (8.3)	11 (3.0)	0.544 (0.261, 1.134,)	0.691 (0.256, 1.866)
*Educational level*				
No formal education	120 (27.5)	54 (15.0)	1	1
Primary school	131 (30.0)	102 (28.3)	1.73 (1.145, 2.614)	1.113 (0.639, 1.939)
Secondary and above	185 (42.4)	205 (56.8)	2.462 (1.688, 3.593)	0.931 (0.518, 1.676)
*Occupational level*				
Housewife	273 (62.6)	189 (52.4)	1	1
Government employee	66 (15.1)	85 (23.5)	1.86 (1.283, 2.696)^∗^	1.223 (0.664, 2.252)
Farmer	60 (13.8)	49 (13.6)	1.18 (0.775, 1.796)	0.969 (0.529, 1.774)
Private employee	37 (8.5)	38 (10.5)	1.483 (0.91, 2.42)	0.979 (0.484, 1.98)
*Number of children*				
No	61 (14.0)	47 (13.0)	1	1
1 to 4	353 (81.0)	306 (84.8)	1.125 (0.747, 1.695)	0.735 (0.412, 1.311)
5 and above	22 (5.0)	8 (2.2)	0.472 (0.193, 1.154)	0.403 (0.117, 1.384)
*Knowledge*				
Poor	405 (92.9)	275 (76.2)	1	1
Moderate	26 (6.0)	65 (18.0)	3.682 (2.278, 5.949)^∗^	1.534 (0.817, 2.881)
Good	5 (1.1)	21 (5.8)	6.185 (2.305, 16.601)^∗^	2.077 (0.651, 6.63)
*Attitude*				
Negative	342 (78.4)	70 (19.4)	1	1
Positive	94 (21.6)	291 (80.6)	15.125 (10.694, 21.392)^∗^	6.164 (4.048, 9.387)^∗∗^
*Subjective norms*				
Negative	331 (75.9)	70 (19.4)	1	1
Positive	94 (21.6)	291 (80.6)	4.005 (2.96, 5.418)^∗^	2.001 (1.342, 2.982)^∗∗^
*PBC*				
Low	311 (71.3)	52 (14.4)	1	1
High	125 (28.7)	309 (85.6)	14.784 (10.318, 21.185)^∗^	7.105 (4.671, 10.807)^∗∗^

^∗^Significantly associated with simple logistic regression analysis. ^∗∗^Significantly associated with multiple logistic regression analysis.

## Data Availability

All the required data has been included in the manuscript

## Abbreviations

AOR: Adjusted odds ratio
CC: Cervical cancer
CCS: Cervical cancer screening
COR: Crude odds ratio
HCP: Health care provider
HPV: Human papilloma virus
PBC: Perceived behavioral control
TPB: Theory of planned behavior
VIA: Visual inspection with acetic acid
WHO: World Health Organization.
